# Cytomegalovirus Anterior Uveitis: Clinical Manifestations, Diagnosis, Treatment, and Immunological Mechanisms

**DOI:** 10.3390/v15010185

**Published:** 2023-01-09

**Authors:** Jing Zhang, Koju Kamoi, Yuan Zong, Mingming Yang, Kyoko Ohno-Matsui

**Affiliations:** Department of Ophthalmology and Visual Science, Graduate School of Medical and Dental Sciences, Tokyo Medical and Dental University, Tokyo 113-8510, Japan

**Keywords:** cytomegalovirus, anterior uveitis, treatment, differential diagnosis, ganciclovir, immunocompetent

## Abstract

Little is known regarding anterior uveitis (AU), the most common ocular disease associated with cytomegalovirus (CMV) infection in immunocompetent populations. CMV AU is highly prevalent in Asia, with a higher incidence in men. Clinically, it manifests mainly as anterior chamber inflammation and elevated intraocular pressure (IOP). Acute CMV AU may resemble Posner–Schlossman syndrome with its recurrent hypertensive iritis, while chronic CMV AU may resemble Fuchs uveitis because of its elevated IOP. Without prompt treatment, it may progress to glaucoma; therefore, early diagnosis is critical to prognosis. Knowledge regarding clinical features and aqueous humor analyses can facilitate accurate diagnoses; so, we compared and summarized these aspects. Early antiviral treatment reduces the risk of a glaucoma surgery requirement, and therapeutic effects vary based on drug delivery. Both oral valganciclovir and topical ganciclovir can produce positive clinical outcomes, and higher concentration and frequency are beneficial in chronic CMV retinitis. An extended antiviral course could prevent relapses, but should be limited to 6 months to prevent drug resistance and side effects. In this review, we have systematically summarized the pathogenesis, clinical features, diagnostic and therapeutic aspects, and immunological mechanisms of CMV AU with the goal of providing a theoretical foundation for early clinical diagnosis and treatment.

## 1. Introduction

Cytomegalovirus (CMV) is a ubiquitous pathogen, and serological tests reveal signs of previous exposure to it in 40% to 100% of the general population [1]. CMV may be transferred horizontally during childhood or adolescence and may also be transferred vertically during pregnancy, leading to systemic manifestations of congenital CMV infection [2,3]. Primary infection with CMV is usually not evident but remains latent in the host, and CMV is able to reactivate when the immune system is compromised [4]. In patients with acquired immunodeficiency syndrome (AIDS), CMV retinitis is the most common opportunistic ocular infection [5]; in the immunocompetent population, CMV infection typically manifests as anterior uveitis (AU) with elevated intraocular pressure (IOP) [6]. On considering the high rate of latent CMV infection, the aforementioned finding may explain the high rate of positivity for CMV AU. However, immunocompromised individuals are less likely to develop CMV AU, which suggests the involvement of a prominent immunological component [7].

CMV AU has been reported worldwide with most cases originating in Asian countries, particularly in Chinese and Japanese populations, which may be associated with their higher seropositivity and genetic susceptibility [8]. While CMV infection can be contracted at any age, CMV AU has a significantly higher prevalence in men aged over 30 years than in other patient groups [9]. Acute CMV AU presents primarily as Posner–Schlossman syndrome (PSS) in individuals aged 30 to 50 years, whereas chronic CMV AU is characterized by Fuchs uveitis (FUS) in individuals aged 50 to 70 years [10], presenting unilaterally in most cases. Clinically, an aqueous humor analysis is frequently used for pathogenic diagnosis, but polymerase chain reaction (PCR) testing does not always serve as an adequate diagnostic test [11]. The difficulty in diagnosing CMV AU can delay the administration of appropriate treatment, which may lead to endothelial dysfunction or permanent corneal damage [12]; further, persistently high IOP could even lead to the requirement of surgical treatment in the case of glaucoma development [13]. Administering antivirals is the primary method of treating CMV infection, but different drug concentrations and administration routes can lead to varying results [14]. In this review, we aimed to systematically summarize the clinical manifestations, diagnostic tests, treatment methods, and immunological mechanisms of CMV AU, with the goal of proposing a theoretical basis for the need for early diagnosis and appropriate treatment administration.

## 2. Clinical Features of CMV AU

CMV AU can be classified as acute CMV AU, chronic CMV AU, recurrent or chronic iridocyclitis, and corneal endotheliitis. Acute CMV AU commonly affects Asian men aged 30 to 50 years and is considered one of the major causes of PSS [15]. The disease is typically characterized by high IOP and mild symptoms in the anterior chamber [16]. The prevalence of chronic CMV AU is high among individuals aged 50 to 70 years, and primarily manifests as ocular discomfort and blurred vision [8]. Iris stroma atrophy can occur as a result [10], particularly in the pupillary region [9]. When a lesion involves the posterior pigment epithelium, it may cause light transmission defects. Occasionally, it is complicated by heterochromia [17]. Unlike Asian patients who present with a syndrome similar to FUS or PSS, Western patients tend to present with a condition that does not have significant clinical features or one that is a combination of FUS and PSS, resulting primarily in recurrent or chronic unilateral iridocyclitis [7]. In corneal endotheliitis, the severity of keratic precipitates (KPs) is consistent with the degree of corneal stromal edema and Descemet’s folds [18]. It may present as a limited or diffuse lesion, may be confined to a single lesion, or may produce severe diffuse corneal stromal edema-like bullous keratopathy [19].

There are several complications associated with CMV AU, including cataracts, corneal disorders, and glaucoma [8]. Persistent IOP elevation may require glaucoma surgery, which is of great concern to patients with CMV AU as it may cause vision loss [20]. Early antiviral interventions can significantly reduce the need for glaucoma surgery; however, this treatment does not prevent further damage in patients with advanced glaucoma [21]. Consequently, an early diagnosis of CMV AU is crucial to achieving favorable prognosis.

## 3. Differential Diagnosis

Diagnosing CMV AU based on clinical signs can be challenging, as CMV belongs to the herpesvirus family and CMV infection can be mistaken for other viral infections such as those caused by herpes simplex virus (HSV), varicella virus (VZV), and rubella virus (RV) [22]. For this reason, this review compares various aspects of these infections (summarized in Table 1) in order to assist in diagnosing CMV AU.

The inflammation caused by CMV AU is usually mild, and comes with associated symptoms such as unilateral headaches, blurred vision, and ocular redness. However, the degree of IOP elevation is inconsistent with the severity of inflammation, and the maximum IOP is higher than that in HSV AU [23]. RV AU is associated with less inflammation [12] and is characterized by Koeppe nodules, whereas HSV AU and VZV AU often produce a severe reaction that presents with acute severe ocular pain, redness, tearing photophobia, and blurred vision [23]. It should be noted that CMV does not produce any cutaneous symptoms, while RV can cause a maculopapular rash [24]; contrarily, VZV infection more commonly manifests as herpes zoster in the distribution of the extended trigeminal nerve, while HSV infection typically presents as marginal blistering with diffuse eyelid edema [25]. In the cornea, CMV infection may result in a decrease in enhanced corneal compensation (ECC); on the other hand, RV infection does not affect the cornea, while HSV and VZV infections may decrease corneal sensitivity or lead to neurotrophic ulcers [26]. HSV causes dendritic ulcers, while VZV causes pseudo-dendritic ulcers, which can be identified by morphological examination and staining [27]. Immune ring keratitis is more commonly seen in HSV AU and VZV AU, but it also occurs in CMV AU [28]. Fan-shaped iris atrophy is most commonly observed in HSV AU and VZV AU, and spiral iris atrophy is usually associated with HSV AU [23]. CMV AU and RV AU do not typically cause posterior iris adhesions with round pupils, while HSV AU and RV AU can result in posterior iris adhesions, leading to eccentric pupil dilation [29].

**Figure 1:**
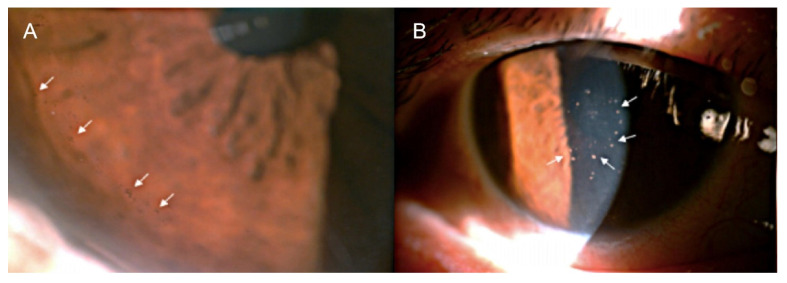
Clinical manifestations of cytomegalovirus anterior uveitis. (**A**) Pigmented keratic precipitates (KPs) (white arrows) in the peripheral cornea. (**B**) Typical coin-shaped KPs (white arrows) in the central cornea (Adapted from Kuo Yu-Wei, et al., Journal of Clinical Medicine, 2022 [30]).

KPs play a vital role in diagnosing infections caused by herpesviruses. Acute CMV AU can present as several medium-sized white or gray KPs distributed centrally or peripherally in the cornea. In Asian patients, chronic CMV AU presents as white or gray stellate (44%) KPs that are diffusely distributed as they are in FUS [12], whereas brown KPs are often seen in chronic CMV AU in Western patients (Figure 1A) [30]. RV AU presents with KPs that are similar to those seen in CMV AU, but does not present with pigmented KPs [7]. It is common for HSV AU and VZV AU to present with small to moderate pigmented KPs in Arlt’s triangle [10].

Despite this broad range of clinical symptoms, some characteristic manifestations deserve attention. A marked decrease in corneal ECC may be a sign of CMV infection, and coin-shaped damage with dusty KPs (Figure 1B) [30] in a ring-like distribution is an important pathological feature of CMV infection (positivity rate, 90.9%) [31]. Iris discoloration is a potential clinical biomarker for predicting chronic or recurrent CMV AU [32]. When clinical manifestations suggestive of CMV AU are observed, pathogenetic analysis should be performed immediately.

## 4. Clinical Diagnostic Testing

Several techniques have been used for pathogenic analysis; we have compared and summarized these techniques in Table 2. Aqueous humor analysis is the most effective method for diagnosing and determining the severity of infection [33]; this analysis is performed by aseptically extracting aqueous humor from the anterior chamber for PCR and Goldmann–Witmer coefficient (GWC) testing, known as detecting deoxyribonucleic acid (DNA) and specific antibody production. As a highly specific and rapid assay, PCR can be used to directly identify the virus [34]. Since PCR can detect very small amounts of DNA, it has a high positive detection rate in the early stages of CMV infection [9]. A qualitative multiplex PCR can be implemented to screen for viruses, and real-time PCR can help in identifying and quantifying viruses and is commonly used for diagnoses and assessments of disease severity [35]. Therefore, real-time PCR can also be used to determine the effects of treatment [36]. False-negative results may occur when viral DNA levels drop below the detection threshold in the late stages of infection, as seen when formulating late-stage diagnoses; a positive PCR test may also be seen if latent viral DNA in anterior chamber leukocytes is detected during the inflammatory phase. Nevertheless, in such cases, CMV is not the causative agent of AU [19].

GWC testing, on the other hand, indirectly determines the presence of infection through the detection of antibodies [37]. Due to the presence of cross-reactive antibodies, GWC testing is less specific than PCR testing. However, GWC testing is more sensitive and thus can help in obtaining a diagnosis when the viral DNA levels are below the PCR detection threshold. Notably, GWC analysis may yield false-negative results because the combination of high serum antibody levels and extensive disruption of the blood–aqueous barrier could mask the positive coefficient [38]. Since diagnostic accuracy depends on the patient’s immune status, degree of infection, and time of sampling, a combined analysis with multiple measurements could reduce the rate of false-positive results and greatly improve diagnostic accuracy [33].

There are methods other than PCR and GWC analyses available. Researchers have calculated the antibody index (AI) to improve diagnostic sensitivity by analyzing intraocular CMV antibody synthesis [39]. Viral cultures can also be used to diagnose viral infections, but the process is difficult and time-consuming. The clinical significance of independent serological tests is limited, with negative results failing to rule out previous viral infection and positive IgM indicating active systemic infection rather than CMV AU [23]. Notably, a new genomic deep sequencing test called metagenomic deep sequencing was developed recently. This test can detect fungi, parasites, and ribonucleic (RNA) and DNA viruses simultaneously, which is promising for the diagnosis of AU [40].

## 5. Currently Used Antiviral Treatments and Updates

After the prompt diagnosis of CMV infection based on clinical symptoms and aqueous humor analysis results, early antiviral treatment should be administered to reduce the risk of the development of glaucoma [13], endothelial dysfunction, and permanent corneal damage [12]. Currently, there is no uniform clinical standard for antiviral therapy for treating CMV AU, and different factors such as drug concentration and administration routes can have a significant impact on treatment effectiveness. Therefore, we have summarized the details of antiviral therapy research in recent years; this summary could serve as a reference to aid in administering the appropriate clinical treatment.

Ganciclovir and valganciclovir inhibit CMV DNA polymerase UL54 through competitive inhibition of viral DNA synthesis [41]. Ganciclovir is commonly used for systemic (intravenous therapy) and topical (intravitreal injection and topical use of eye drops or gel) antiviral therapy, while valganciclovir is commonly used for systemic therapy (oral therapy). Systemic therapy is more effective in controlling inflammation than topical therapy [42], possibly because a higher therapeutic concentration of ganciclovir is maintained in the aqueous humor as a result of systemic therapy [43]. Systemic therapy includes oral and intravenous treatments, both of which are effective in controlling inflammation and reducing IOP [13]. Although the side effects in immunocompetent people are usually mild, systemic therapy requires regular blood tests to assess complete blood counts and renal function. Moreover, considering the high cost of systemic treatment doses, topical ganciclovir administration is a relatively economical option. However, some investigators have reported on the threat of infection and retinal tears and detachment associated with vitreous injections and still recommend the use of systemic therapy as the first-line treatment option [13]. It has been demonstrated that the level of systemic toxicity is lower when using intravitreal ganciclovir (2 mg/0.05 mL), and the option of using this treatment as an alternative to systemic ganciclovir has been explored [14]. On administering a vitreous injection of ganciclovir, a high ganciclovir concentration was maintained in the vitreous humor (600 ng/mL) for 7 days [44], but CMV DNA was not completely eliminated from the aqueous humor even after four weekly injections [45]. These results may explain the high recurrence rate after the discontinuation of treatment with intravitreal ganciclovir injections [42]; therefore, many investigators believe that topical/oral antiviral therapy should be continued after cessation of treatment with intravitreal injections.

Regarding treatment with eye drops or gel dosing, it is recommended that ganciclovir concentrations range between 0.15% and 2% and that the medication be applied six to eight times daily during induction and one to four times daily during maintenance [46,47]. According to Langston et al., frequent dosing (2 h/dose) of ganciclovir gel resulted in better efficacy [48]. Based on Antoun’s study, 0.15% ganciclovir gel produces the best therapeutic effect, although patient compliance was not high [49]. Therapy with 2% ganciclovir drops yielded effective results in the case of CMV endotheliitis and AU. Moreover, 0.15% ganciclovir gel has minimal toxicity and is well tolerated by patients. In addition to topical treatment, oral valganciclovir is also a common clinical treatment. It has been reported that 900 mg of oral valganciclovir taken two times daily was effective in preventing glaucoma surgery in more than one-third of patients with CMV AU with uncontrolled IOP within 2 years [50]. Other studies have demonstrated that oral valganciclovir is effective in controlling IOP and reducing the need for glaucoma surgery [51,52]. It may be more feasible to treat patients with topical agents over the long term because of their low toxicity, owing to which laboratory monitoring is not required.

The high frequency of CMV AU recurrence has led some investigators to recommend an extended course of antiviral therapy (>12 months) [43]; however, other have suggested that long-term use leads to CMV-resistant strains and systemic side effects [14]. Therefore, it is recommended that maintenance therapy should not exceed 6 months in order to control CMV virus replication while reducing the risk of relapse [13]. Leukopenia and neutropenia are adverse events induced by valganciclovir in immunocompetent patients, while abnormal renal function and gastrointestinal discomfort are side effects [50]. Topical ganciclovir gel at a concentration of 0.15% is less toxic and generally well tolerated, although some patients have reported mild ocular discomfort. Prolonged and intensive use of ganciclovir gel may cause epithelial cell toxicity [46]. Therefore, patients should be carefully monitored during the course of antiviral therapy to avoid adverse events.

In cases in which ganciclovir/valganciclovir therapy leads to severe side effects or produces no therapeutic effects, letermovir may be a suitable alternative. Letermovir was approved by the Food and Drug Administration (FDA) for the prevention of CMV infection in patients who received hematopoietic stem cell transplantation (HSCT). Its underlying mechanism is to inhibits viral replication via the CMV terminase complex (UL56) [53], and studies have shown that 480 mg of letermovir daily reduces inflammation in CMV AU [54]. Furthermore, letermovir does not induce myelosuppression or nephrotoxicity, is well tolerated by patients, and does not require laboratory monitoring. It should be noted that letermovir is more likely to cause resistance than ganciclovir or foscarnet [55]. Therefore, letermovir might only be indicated for short-term use.

In addition to letermovir, patients who are resistant or intolerant to ganciclovir may benefit from CMV immunoglobulin (CMV IG) [56]. CMV IG was approved by the US FDA as a prophylactic anti-CMV treatment for high-risk lung transplant recipients, and a study found that 14 of 15 recipients with acute CMV infection following cardiothoracic transplantation could be converted to negative after two doses. Although a higher rate of adverse obstetric events was reported in the CMV IG treatment group [57], there was no statistical difference owing to the low number of CMV-infected pregnant women. In contrast, other studies have reported support for the safety of CMV-specific immunoglobulins and efficacy in preventing CMV infection and disease [58,59,60,61]. Therefore, further research is needed before drawing conclusions on whether CMV IG is recommended for pregnant women. Notably, there are no supporting data regarding the use of CMV IG in the treatment of CMV AU. Thus, further research is needed to examine the efficiency of this therapy.

## 6. Currently Known Immune Mechanisms and Updates

Knowledge regarding the immune mechanisms underlying CMV infection could contribute to a better understanding of its clinical features and to developing new therapeutic options. CMV is capable of infecting various types of cells; following infection, the CMV genome enters the nucleus and starts viral gene transcription, thereby producing infectious particles [62]. The main defense mechanism against host infection with CMV is innate and adaptive immunity, and natural killer (NK) cells are considered to act as a bridge between these two types of immunity [6]. When T cells are activated, they multiply and differentiate into cytokine-secreting effector T cells that destroy CMV-infected cells to limit viral spread and clinical symptoms [63]. CMV-specific CD8+ T cells dominate cellular immunity during primary infection [64]. CMV-specific CD4+ T cells promote the recruitment of CD8+ T cells to the site of infection by secreting chemokines that stimulate the expansion and differentiation of CD8+ T cells [65]. However, in persistent infections, CMV-specific CD4+ T cells play a dominant role by producing antiviral effects through the production of cytokines, interferon gamma (IFN-γ), and tumor necrosis factor (TNF), as well as through direct cytolysis with perforin- and Fas-dependent killing [66]. Therefore, adaptive immunity is crucial for controlling CMV latency and reactivation, with CD4+ and CD8+ T cells playing essential roles in controlling the immune response. These findings have led to CMV-specific T cells becoming a hot topic for research in recent years, with some studies explaining the specific clinical manifestations of CMV.

CMV AU is known to differ significantly based on patient sex [16], which may be a result of the pro-inflammatory response to CD4+ and CD8+ T cells being greater in men than in women [67]. As for immune evasion in CMV AU, several studies have suggested that it is related to the fact that IE, E, and L genes are transcribed in a stochastic manner during latent infection [68], evading the immune response by encoding multiple evasion proteins [69]; others propose that the cytotoxic T cell response disappears through the downregulation of the human leukocyte antigen (HLA)-A and HLA-B when the host is still able to respond through NK cells, leading to the absence of the cytotoxic T cell response [70]. The high rate of CMV AU relapse may be attributed to latent CMV inhibiting the antiviral activity of CD4+ T cells by regulating CD14+ monocyte secretion, which leads to the reactivation of CMV [71]. Considering that T cell function determines the control of CMV infection, recent studies have concluded that infusions of CMV-specific T cells can transfer protective immunity [72,73,74].

Immunocompetent populations are most likely to develop CMV AU, which is also associated with CMV-specific T cells. Using an established mouse CMV model, investigators verified that systemic CMV infection in immunocompetent hosts leads to an intense inflammatory response and latency in the anterior ocular segment [75]. After systemic CMV infection, viral antigen staining revealed that the virus could enter and replicate in the iris, ciliary body, choroid, and cornea. This suggested that CMV infection leads to the infiltration and accumulation of antiviral CD8+ T cells in the eye, leading to the development of tissue resident memory T cells (TRM). Even on day 250, large numbers of CD45+ cells were detected in the frontal lobe, which indicated latent infection. After controlling the viral infection, foci of giant cells and viral cells were detected in iris explants, demonstrating that CMV can cause latent infection in the eye. As CMV is a virus capable of latently inducing long-term inflammation of the eye, CMV AU, the most common ocular symptom in immunocompetent populations, deserves more attention and requires early intervention.

## 7. Conclusions

In immunocompetent populations, CMV AU is the most common ocular disease that develops following CMV infection, and it involves the inflammation of the anterior segment of the eye. Untreated CMV AU may progress to iris atrophy and glaucoma, which makes prompt diagnosis and treatment essential for achieving a favorable prognosis. Clinical diagnosis can be made quickly and accurately by aqueous humor analysis, but multiple measurements combined with different analyses are required to avoid false results. Early antiviral therapy could significantly reduce the risk of glaucoma surgery, but there is a need for further standardization of uniform antiviral treatment criteria for CMV AU. Current antiviral therapies include intravenous and topical (intravitreal injections and ophthalmic drops) ganciclovir and oral valganciclovir. For patients who are intolerant to their side effects, letermovir is available and CMV IG is promising but needs further research. According to immune mechanism studies, specific T cells play pivotal roles in controlling infection. Further immune studies are needed to better understand the clinical features and develop new therapeutic options. The fact that CMV can cause latent infection in the eye and can induce long-term inflammation warrants more attention paid to the early diagnosis and timely treatment of CMV AU.

## Figures and Tables

**Table 1:** Comparison of clinical features between common viral anterior uveitis etiologies.

Variable ^1^	HSV	VZV	CMV	RV
Age (years)	<50	>60	Acute, >30 chronic, >50	20–40
Sex	No predilection	No predilection	Male predilection	No predilection
Race	All	All	Asian	Western
Symptoms	Severe	Severe	Mild or absent	Mild or absent
Intraocular pressure	Acute spikes	Acute spikes	Very high (Up to 50 mmHg)	Persistent elevation
Keratic precipitates				
Distribution	Arlt’s triangle	Arlt’s triangle	Diffusely distributed	Diffusely distributed
Morphology	Small to medium	Small to medium	Medium to large, coin shaped	Fine stellate and non-stellate
Color	Pigmented	Pigmented	White or pigmented	White
Dermal manifestation	Vesicular rash	Dermatomal rash	None	Maculopapular rash
Corneal	Dendritic ulcers	Pseudodendritic ulcers	Endotheliitis, endothelial cell loss	Normal
Iris atrophy	Sectoral iris atrophy	Spiral atrophy	Iris stromal atrophy	Diffuse atrophy
Pupil	Eccentric dilated	Eccentric dilated	Round	Round
Cataract	Late at presentation	Late at presentation	Late at presentation	Early at presentation
Vitritis	Always	Sometimes	Rarely	Always

^1^ Abbreviations: CMV, cytomegalovirus; HSV, herpes simplex virus; VZV, varicella virus; RV, rubella virus.

**Table 2:** Comparison of techniques for pathogenic analysis.

Technique *	Sensitivity	Specificity	Feasibility	Rapidness	Application
PCR	++	+++	+++	+++	Early-stage diagnosis
Qualitative multiplex PCR	++	+++	+++	+++	Virus screening
Real-time PCR	++	+++	+++	+++	Detection of viral loads
GWC	+++	+	+++	+++	Late-stage diagnosis
Viral culture	+	+++	+	+	Accurate diagnosis
Isolated serological tests	+	+	++	+++	Limited value
Metagenomic deep sequencing	+++	+++	+	+++	Detection of fungi, parasites, and DNA and RNA viruses simultaneously

* Sensitivity: probability of detecting true positives. Specificity: probability of detecting true negatives. Feasibility: practicability in routine analysis, execution, and interpretation. The number of + symbols represent the rating of the methods in each criterion from acceptable (+), normal (++) to optimum (+++). Abbreviations: PCR, polymerase chain reaction; GWC, Goldmann–Witmer coefficient; DNA, deoxyribonucleic acid; RNA, ribonucleic acid.

## Data Availability

All data related to this study are presented and published here.
